# Prevalence and associated factors of postoperative orthostatic intolerance at University of Gondar Comprehensive Specialized Hospital, Northwest Ethiopia, 2022: cross sectional study

**DOI:** 10.1186/s12893-023-02015-5

**Published:** 2023-05-01

**Authors:** Negesse Zurbachew Gobezie, Nigussie Simeneh Endalew, Hailu Yimer Tawuye, Habtu Adane Aytolign

**Affiliations:** 1 Department of Anesthesia, College of Medicine and Health Sciences, Debre Tabor University, Debre Tabor, Ethiopia; 2 Department of Anesthesia, College of Medicine and Health Science, University of Gondar, Gondar, Ethiopia

**Keywords:** Surgery, Postoperative, Prevalence, Factors, Orthostatic intolerance, Ambulation

## Abstract

**Background:**

Postoperative orthostatic intolerance is an inability to maintain an upright position because of symptoms of cerebral hypoperfusion. It is a common problem in the early postoperative period and hinders early mobilization, however, there is limited information about factors associated with it. Thus, the main aim of this study was to determine the prevalence and identify factors associated with postoperative orthostatic intolerance.

**Method:**

Hospital based cross-sectional study was conducted from April 08 to July 20, 2022, at University of Gondar comprehensive Specialized Hospital. A semi-structured questionnaire containing sociodemographic variables and perioperative factors related to anesthesia and surgery was used for data collection. The presence of postoperative orthostatic intolerance during the first ambulation was evaluated with a standardized symptom checklist which contains symptoms of orthostatic intolerance. Binary logistic regression analysis was performed to assess factors associated with postoperative orthostatic intolerance. In multivariable regression, variables with *P*-value < 0.05 were considered statistically significant.

**Result:**

A total of 420 patients were included in this study with a response rate of 99.06%. Postoperative orthostatic intolerance was experienced in 254 (60.5%) participants. Being female (AOR = 2.27; 95% CI = 1.06–4.86), low BMI (AOR = 0.79; 95% CI = 0.71–0.95), ASA II and above (AOR = 3.34; 95% CI = 1.34–8.28), low diastolic blood pressure (AOR = 0.82; 95% CI = 0.88–0.99), general anesthesia (AOR = 3.26, 95% CI = 1.31–8.12), high intraoperative blood lose (AOR = 0.93, 95% CI = 0.88–0.99), high postoperative fluid intake (AOR = 2.09, 95% CI = 1.23–3.55), pain before ambulation (AOR = 1.99, 95% CI = 1.28–3.11) and pain during ambulation (AOR = 1.82, 95% CI = 1.23–2.69) were the significant factors associated with orthostatic intolerance.

**Conclusion:**

Our study revealed that postoperative orthostatic intolerance was experienced in nearly two-thirds of participants. During the time of ambulation, assessing patients for the presence of orthostatic intolerance is necessary to reduce the adverse effects of postoperative OI. In addition, maintaining preoperative normotension, reducing intraoperative blood loss and optimizing postoperative pain control is recommended to reduce the risk of postoperative orthostatic intolerance.

## Background

Orthostatic intolerance is an inability to maintain an upright posture due to symptoms of cerebral hypoperfusion, such as lightheadedness, headache, nausea, vomiting, feeling of heat, blurred vision, and eventually syncope [1, 2].

Due to the effect of gravity during maintaining an upright position around 500–700 ml of blood will be displaced from the upper part of the body to the lower parts of the body into the abdominal and lower extremity vasculature [3, 4]. Thus, upright posture results in a decrease in central blood volume and cardiac output, which may lead to hypoperfusion of the brain. However, in normal physiology, the gravity induced reduction in venous return and brain perfusion is counteracted and maintained by a reflex increase in total peripheral resistance [5, 6], activation of the muscle pump, and vasoconstriction of capacitance vessels [7].

Blood and fluid loss during surgery can exacerbate the postural reduction in central blood volume and blood flow to the brain that occurs in the upright position [8, 9]. In addition, medications used for premedication, anesthesia, and analgesia including opioids, can reduce arterial pressure, cerebral blood flow and oxygenation and contribute to orthostatic intolerance (OI) [5, 6].

Moreover, systemic inflammatory syndrome secondary to surgical stress response also triggers reflex parasympathetic activity and results in impairment of reflex vasoconstriction of capacitance vessels [10]. Due to these reasons, surgical patients may be especially vulnerable to OI during postoperative mobilization. Pain-induced impairment in vasomotor response, residual effects of anesthesia and anemia also contribute to the development of postoperative OI [11].

Postoperative OI is a common problem immediately after surgery which affects up to 60% of patients in the early postoperative period and hinders implementation of the modern fast-track surgery patient care [11–13].

Nowadays, a multimodal approach fast-track surgery, also known as enhanced recovery after surgery (ERAS) programs, has been developed and incorporated into patients’ surgical care pathway and is widely recommended for every surgical procedure to accelerate patient recovery and reduce postoperative complications [14–16]. Early postoperative mobilization is one of the central components of the modern perioperative care program for surgical patients. According to the ERAS program, surgical patients are advocated to perform post-operative mobilization activities as early as 2 h postoperatively [16, 17]. Early mobilization after surgery is important to prevent a wide range of postoperative complications, accelerate patient recovery and reduce the length of postoperative hospital stay and hospital costs [18–20].

Despite the improved management of pain and other major postoperative complications that may affect earlier mobilization, early mobilization after surgery remains challenging because of postoperative OI, which tends to prolong hospital stay after minor ambulatory [1, 21] and major surgical procedures [8, 11]. Delayed postoperative ambulation due to the OI increases the risk of deep venous thrombosis, pulmonary embolism, pneumonia, atelectasis, urinary tract infections, sepsis, myocardial infarction and stroke [18, 20].

In addition, post operative OI is one of the causes of non-cardiogenic syncope and can cause postoperative syncope which leads to potential serious complications such as falls and life-threatening trauma [3, 11, 22]. In bariatric surgeries, OI can be severe and may need vasopressor support and it can persist for a longer time, up to 5-year period after surgery and affects postoperative long-term quality of life [23, 24].

Despite the fact that OI is a common postoperative problem, few studies have investigated potential risk factors for developing OI during early postoperative mobilization [11]. Studies examining the potential risk factors for postoperative OI are limited and the existing studies are conducted with small sample size in specific group of surgical procedures and they recommend further studies to be conducted using a larger sample size and more diverse patient population [11, 25]. Therefore, this study was conducted to fill the information gap regarding the prevalence and associated factors of postoperative OI. Determining the prevalence and its associated risk factors of postoperative OI among surgical patients will provide an important and up-to-date information on the prevalence and major predictor factors of postoperative OI.

In addition, the study will help to identify major factors associated with postoperative OI and can provide valuable information for health care providers that participate in perioperative patient care and help to improve perioperative patients’ care and will help to reduce the perioperative patient morbidity.

## Methods and materials

### Study design, period and settings

Hospital based cross-sectional study was conducted on adult surgical patients that underwent surgery under anesthesia in UOGCSH from April 08, 2022 to July 20, 2022. This hospital is located in Amhara region, Central Gondar administrative zone, which is about 750 km away from Addis Ababa (the capital city of Ethiopia) in Northwest of Ethiopia [26]. This hospital is a teaching hospital which serves more than seven million people of the Central Gondar zone and peoples of the neighboring zones. According to the UOGCSH planning and program coordination office report, more than six thousand patients were operated on under anesthesia per annum.

### Study population

All consequtive adult surgical patients (18 years and above) who were operated under anesthesia upon emergency and elective conditions and were admitted in post anesthesia care unit (PACU), surgical, orthopedic, and gynecologic wards were included. Patients who had neuropsychiatric problems that impairs their communications, patients who were unable to ambulate in postoperative period, patients who are admitted to ICU after operation, obstetric mothers, headinjury patients were excluded from the study.

### Sample size determination

The actual sample size for the study was determined by using single population proportion formula. $n=\frac{{\left(Z{}^{\alpha }\!\left/ \!{}_{2}\right.\right)}^{2}\rho \left(1-\rho \right)}{{\varepsilon }^{2}}$. Where n = sample size Z = the standard normal value at the level of confidence desired, $\rho$ = proportion, $\varepsilon$= marginal error. Since there is no previous similar study conducted in the study area and in Ethiopia, the proportion (prevalence) is assumed as 50%. By assuming a 95% of confidence interval with a 5% margin of error, *p* = 0.5 and, $\varepsilon$  = 5%, zα/2 = 1.96.

$$\frac{\left(1.96\right)2 * 0.5\left(1-0.5\right)}{\mathrm{n}=(0.05)2}$$

n = 384.16 ≈ 385. Adding 10% non-response rate the final sample size was 424.

### Variables

The outcome variable of this study was prevalence of postoperative OI. Age, sex, BMI, ASA physical status, preoperative medication intake, preoperative haemoglobin, preoperative blood pressure, comorbidities, urgency of surgery, grade of complexity of surgery, duration of anaesthesia, duration of surgery, amount of intraoperative blood loss, amount of intraoperative fluid given, intraoperative and postoperative opioid administration, pain score before ambulation (at rest) and pain score during mobilization were independent variables of this study.

### Operational definition

**Postoperative OI** was defined as appearance of one of the following presyncope symptoms: dizziness (light headedness), nausea, vomiting, feeling of heat, headache, blurred vision and ultimately syncope at the time of first ambulation during postoperative period [27, 28].

**Time of first ambulation** was defined as time from end of surgery and anesthesia until patient ambulate at first time.

### Data collection procedure

A semi-structured questionnaire was prepared to address sociodemographic, preoperative, intraoperative and postoperative variables associated with postoperative OI. The data collection procedures were through reviewing of the chart and interviewing of the participants. Preoperative, intraoperative and postoperative clical variables were collected from chart review. Grade of complexity of surgery was classified into minor, intermediate and major surgery based on NICE guideline grade of complexity of surgery classification [29]. Pain before ambulation (at rest) and pain during the first ambulation was assessed by using 11 point numeric rating pain scale (NRS 0 to 10, 0 = no pain, 10 = worst pain ever). Presence of postoperative OI at the time of first ambulation was evaluated with a standardized symptom checklist, developed in previous studies [27]. Based on the defined symptoms, the patient was considered to have OI if the patient developed one or more symptoms of cerebral hypoperfusion (i.e., lightheadedness, nausea, vomiting, feeling of heat, headache, blurred vision and ultimately syncope) during first ambulation.

### Data quality control

Pre-test of the data collection was done on 22 participants (5% of the calculated sample size) at UOGCSH two weeks before actual data collection time to check questionnaire validity. The data collectors and data collection supervisors were trained how to approach study subjects; how to use the data collection tools and how to collect data from patients. During data collection regular supervision and follow up were done for the completeness, accuracy and clarity of data.

### Data management and statistical analysis procedures

After completion of data collection, the variables were coded and entered using Epi-data software (version 4.6) and exported into Stata version 14 software for analysis. Patients were grouped by those experiencing OI and those who did not experience OI and coded as 1 and 0 respectively. Continuous variables were evaluated for normal distribution using Shapiro-Wilks normality test. Categorical variables are reported as number (%) and continuous variables reported as median with IQR. Both bivariable and multivariable binary logistic regression analysis was performed to assess the strength of association between dependent and independent variables. Independent variables with *P* < 0.20 at 95% CI in the bivariable analysis were fitted to the multivariable binary logistic regression analysis. In multivariable regression, variables with *P*-value < 0.05 were considered as statistically significant associated factors for postoperative OI. Crude odds ratio (COR) and adjusted odds ratio (AOR) with the corresponding 95% confidence interval was calculated to determine the strength of association of independent factors with outcome variable (i.e., OI). Model fitness was checked using Hosmer and Lemeshow goodness of fit test and was not significant (*p* = 0.433). Multicollinearity for multivariable binary logistic regression analysis was checked using the variance inflation factor and found that there was no multicollinearity since all variables had variance inflation factor < 10 and tolerance greater than 0.1.

## Result

During the study period, a total of 424 patients who fulfilled the inclusion criteria were included. A total of 420 participants (48 gynecologic surgeries, 68 orthopedic surgeries and 304 general surgery) were included in final analysis. Four patients were excluded from the analysis due to incomplete data making a response rate of 99.06%.

### Sociodemographic and preoperative clinical variables of study participants

From a total of 420 study participants, 217 (51.8%) were male, and the median (IQR) age was 34 (27–47) year. More than half of the study participants (58.02%) were ASA physical status I. From the total study participants, 11 (2.62%) had DM, 30 (7.14%) had hypertension and 8 (1.90%) had cardiovascular disease. From the total participants, 61 (14.52%) took beta blocker medications preoperatively and 5.95% of participants took tramadol preoperatively (Table 1).

**Table 1:** Sociodemographic characteristics and preoperative clinical variables of study participants in UOGCSH, 2022

	Frequency (%) or Median IQR		
		Postoperative orthostatic intolerance	
Variable	Total (*N* = 420)	No (*n* = 166)	Yes (*n* = 254)
Gender^a^			
Male	217 (51.67)	99 (45.62)	118 (54.38)
Female	203 (48.33)	67 (33.0)	136 (67.0)
Age (years)^b^	34 (27–47)	32.5 (26–45)	35.5 (27–48)
BMI (Kg/m^2^)^b^	20.2(18.36- 21.99)	21.28 (19.72–22.49)	19.38 (17.63–20.82)
ASA physical status^a^			
ASA I	246 (58.57)	127 (51.63)	119 (48.37)
ASA II and above	174 (41.43)	39 (22.41)	135 (77.59)
Preop Hemoglobin (g/dl)^b^	13.6 (12.5- 15)	14 (12.8- 15)	13.5(12- 14.6)
Preoperative SBP^b^	120 (110–130)	120 (110–130)	120 (110–130)
Preoperative DBP^b^	70 (65.5- 80)	71 (70- 80)	70 (65- 80)

*Abbreviations*: *IQR* Inter quartile range, *N* Number, *SBP* Systolic blood pressure, *DBP* Diastolic blood pressure, *g/dl* Grams per deciliter, *Kg/m*^*2*^ Kilograms per meter square

Categorical variables (^a^) are expressed in frequency (%) and continuous variables (^b^) are expressed as median (IQR)

### Intraoperative and postoperative variables of study participants

Among the total participants, 286 (68.1%) were elective patients. From the total participants, 202 (48.1%) underwent major surgery, 155 (36.9%) intermediate and 63 (1.5%) minor surgery. In terms of anesthesia type, 275 (65.5%) participants were operated under general anesthesia. Intraoperative opioid was administered to 268 (63.8%) of the participants. The median (IQR) postoperative pain score during rest and during ambulation was 4 (2–6) and 6 (4–8) respectively. The median duration of surgery was 100 min with an IQR (60–120 min (Table 2).

**Table 2:** Intraoperative and postoperative variables of study participants in UOGCSH, 2022

	Frequency (%) or Median IQR		
		Postoperative orthostatic intolerance	
Variable	Total (*N* = 420)	No (*n* = 166)	Yes (*n* = 254)
Urgency of surgery^a^			
Emergency	134 (31.90)	41 (30.60)	93 (69.40)
Elective	286 (61.10)	125 (43.71)	161 (56.29)
Grade of complexity of surgery^a^			
Minor	63 (15.0)	43 (68.25)	20 (31.75)
Intermediate	155 (36.90)	60 (38.71)	95 (61.29)
Major	202 (48.10)	63 (31.19)	139 (68.81)
Type of anesthesia^a^			
Regional	145 (34.52)	88 (60.69)	57 (39.31)
General	275 (65.48)	78 (28.36)	197 (71.64)
Estimated Blood loss (ml)^b^	250 (100–400)	150 (100- 320)	300 (150–500)
Intraoperative fluid (L)^b^	2 (1–2)	1 (1.5- 2)	1 (2–2)
Postoperative fluid (L)^b^	2 (1- 2)	1 (1- 2)	2 (1.5—3)
Resting pain in NRS^b^	4 (2- 6)	2 (1- 3)	5 (3—6)
Pain during first ambulation^b^	6 (4—8)	4 (3—6)	7 (6—8)

*Abbreviations*: *IQR* Inter quartile range, *N* Number, *L* Liter, *min* Minute, *ml* Milli-liter, *EBL* Estimated blood loss, *NRS* Numeric rating scale

Categorical variables (^a^) are expressed in frequency (%) and continuous variables (^b^) are expressed as median (IQR)

### Prevalence of postoperative OI

The median (IQR) time of postoperative first ambulation was 11 (8–16) hours. At this time point, 254 patients demonstrated OI making the prevalence of postoperative OI 60.50% (95% CI = (55.62–65.18%)). Symptoms associated with OI were as follow. Lightheadedness was experienced in 91 (35.83%) participants. Nausea and vomiting presented in 87 (34.25%) and 41 (16.14%) participants respectively. Feeling of heat was complained by 42 (16.54%) participants. Headache and blurred vision presented in 41 (16.4%) and 75 (29.5%) respectively. Syncope appeared in 6 (2.36%) of participants.

### Factors associated with postoperative OI

Using bivariable analysis gender, ASA physical status, urgency of surgery, grade of complexity of surgery, anesthesia type, postoperative opioid use, age, BMI, preoperative hemoglobin, preoperative SBP, preoperative DBP, intraoperative fluid given, intraoperative EBL, postoperative fluid, postoperative pain before ambulation and postoperative pain during ambulation were found to be significant with P < 0.20. Subsequently, these variables were fitted to multivariable analysis and gender, BMI, preoperative DBP, ASA status, anesthesia type, intraoperative EBL, postoperative fluid, pain before ambulation (at rest) and pain during ambulation were significantly associated with postoperative OI.

Being female increases the risk of developing postoperative OI 2.27 times as compared to male (AOR = 2.27, 95% CI = (1.06–4.86)). One unit increase in BMI in Kg/m^2^, decreases the odds of developing postoperative OI by 18% (AOR = 0.82, 95% CI = 0.71–0.95). More over one unit increase in intraoperative blood loss in ml increases the odds of postoperative OI by 0.3% (AOR = 1.003, 95% CI = 1.001–1.010). The odds of postoperative OI among ASA II and above patients is 3.34 times higher as compared to ASA I patients (AOR = 3.34, 95% CI = 1.34–8.28). One unit increase in preoperative DBP decreases the risk of postoperative OI by 7% (AOR = 0.93, 95% CI = 0.88–0.99). Regarding the type of anesthesia, being operated under general anesthesia increases the risk of postoperative OI by 3.26 times as compared to regional anesthesia (AOR = 3.26, 95% CI = 1.31–8.12). Moreover, a unit increase in postoperative fluid intake in liter increases the odds of developing postoperative OI by a factor of 2.09 (AOR = 2.09, 95% CI = 1.23–3.55). Regarding to post operative pain, one unit increase in resting pain and pain during first ambulation increases the odds of postoperative OI by 99% (AOR = 1.99, 95% CI = 1.28–3.11) and 82% (AOR = 1.82, 95% CI = 1.23–2.69) respectively (Table 3).

**Table 3:** Factors associated with postoperative OI: result from bivariable and multivariable binary logistic regression analysis in UOGCSH, 2022

Variables	Postoperative OI (*N* = 420)		OR (95% CI)		
	No [n (%) or median (IQR)]	Yes [n (%) or median (IQR)]	COR (95% CI)	AOR (95% CI)	*P* value
Age (years)^b^	32.5 (26–45)	35.5 (27–48)	1.0 (0.99–1.03	1.02 (0.99–1.05)	0.138
Gender^a^					
Male	99 (45.62)	118(54.38)	1	1	
Female	67 (33.0)	136 (67.0)	1.70 (1.15–2.53)	2.27 (1.06–4.86)	**0.035**
BMI^b^	21.3(19.72–22.49)	19.4(17.63- 20.8)	0.77 (0.70–0.84)	0.82 (0.71–0.95)	**0.006**
ASA status^a^					
ASA I	127 (51.63)	119 (48.37)	1	1	
≥ ASA II	39 (22.41)	135 (77.59)	3.69 (2.39–5.70)	3.34(1.34–8.28)	**0.009**
Hemoglobin^b^	14 (12.8–15)	13.5 (12- 14.6)	0.86 (0.77–0.96)	0.91 (0.76–1.10)	0.329
Preop SBP^b^	120 (110–130)	120 (110–130)	0.99 (0.98–1.01)	1.04 (0.99–1.08)	0.099
Preop DBP^b^	71 (70- 80)	70 (65- 80)	0.97 (0.95–0.99)	0.93 (0.88–0.99)	**0.040**
Urgency of surgery^a^					
Elective	125 (43.71)	161 (56.29)	1	1	
Emergency	41 (30.60)	93 (69.40)	1.76 (1.14–2.72)	0.41 (0.14–1.18)	0.10
Grade of surgery^a^					
Minor	43 (68.25)	20 (31.75)	1	1	
Intermediate	60 (38.71)	95 (61.29)	3.40 (1.83–6.34)	1.16 (0.37–3.69)	0.797
Major	63 (31.19)	139 (68.81)	4.74 (2.58–8.72)	0.28 (0.07–1.11)	0.070
Type of anesthesia^a^					
Regional	88 (60.69)	57 (39.31)	1	1	
General	78 (28.36)	197 (71.64)	3.89 (2.55–5.96)	3.26 (1.31–8.12)	**0.011**
Intraoperative EBL (ml)^b^	150 (100- 320)	300 (150–500)	1.003 (1.002–1.005)	1.003 (1.001–1.010)	**0.011**
Postoperative opioid used^a^					
Yes	52 (25.87)	149 (74.13)	3.11 (2.06–4.69)	0.47 (0.20–1.08)	0.075
No	114 (52.05)	105 (47.95)	1	1	
Postop fluid (L)^b^	1 (1–2)	2 (1.5- 3)	3.42 (2.51–4.68)	2.09 (1.23–3.55)	**0.007**
Pain at rest^b^	2 (1- 3)	5 (3- 6)	2.64 (2.21–3.15)	1.99 (1.28–3.11)	**0.006**
Pain during ambulation^b^	4 (3- 6)	7 (6–8)	2.65(2.22- 3.16)	1.82 (1.23–2.69)	**0.003**

*Abbreviations*: *AOR* Adjusted odds ratio, *COR* Crude odds ratio, *CI* Confidence interval, *IQR* Inter quartile range, *n* Number, *ASA* American society of anesthesiologist, *BMI* Body mass index, *SBP* Systolic blood pressure, *DBP* Diastolic blood pressure, *EBL* Estimated blood loss, *OI* Orthostatic intolerance, *preop* Preoperative

Categorical variables (^a^) are expressed in frequency (%) and continuous variables (^b^) are expressed as median (IQR)

*P* values < 0 .05 are considered significant (bold)

## Discussion

Our study found that as many as 60.5% of patients experienced OI during the time of first postoperative ambulation. The result of this study is in line with the study conducted in Denmark (60%) [28]. On the other hand, the result of this study is considerably high as compared to studies conducted in New Zealand (22%) [27], Japan (35.2%) [8] and in Colorado (18%) [25]. The reason for this discrepancy may be due to the inclusion of diverse group of patients with different patient characteristics and due to large sample size in our study.

The result of our study showed that low BMI is significantly associated with postoperative OI. This finding is also supported by a previous study conducted in Denmark [28]. The existing evidence suggests that the association might be due to low stroke volume secondary to cardiac atrophy and hypovolemia in patients with low BMI and it was also due to an up-regulation of parasympathetic nervous system activity and downregulation of sympathetic nervous system activity in underweight individuals that may lead to impaired compensatory mechanism during mobilization in postoperative period and can cause OI syndrome [11, 28]. It also supposed that BMI is positively correlated with total peripheral resistance and mean blood pressure. Patients with low BMI have low total peripheral resistance and reduced blood pressure during upright position will predispose to orthostasis [30].

In our study female sex is significantly associated with postoperative OI. Our finding is also supported by studies conducted in Korea [6], Denmark [28], Japan [8] and New Zealand [27]. The existing evidences stated that this is due to the presence of sex differences in autonomic function and blood pressure regulation. Male individuals can immediately sustain increase in sympathetic activity during an orthostatic challenge, whereas the increment in sympathetic activity during orthostatic challenge is minimal and delayed in onset in female individuals. It is also supposed that female individuals have increased lower limb compliance that leads to venous pooling during upright position and leads to decrement in central blood volume during upright position [27].

Our study also revealed that being ASA class II and above was significantly associated with postoperative OI. Our finding was supported by previous study conducted in laparoscopic colorectal resection patients [28]. In contrast to this, in other previous studies ASA physical status is not associated with postoperative OI [8, 22, 31]. The reason for this inconsistency might be due to the exclusion of patients with higher ASA physical status (above ASA II) from their study. It was supposed that patients with higher ASA physical status have more comorbidities and they are aged that causes impaired vasomotor reflex and leads to orthostasis [25].

Even though different studies postulated that the presence of number of coexisting illnesses like DM, hypertension and cardiovascular disease and preoperative intake of antihypertensive medications will increase the risk of postoperative OI [11, 27], in our study no factors from patient medical history or preoperative medications were associated with postoperative OI. This may be due to presence of few numbers of participants with preexisting comorbidities in our study which leads to difficulty to determine the association between preexisting comorbidities and postoperative OI.

Even though preoperative DBP has no significant association with postoperative OI in few studies [12, 25], our study revealed that low preoperative DBP is significantly associated with postoperative OI. The reason for this difference might be inclusion of diverse group of patients including, emergency and trauma surgeries in our studies. Previous studies used specific types of surgical procedures and there may not be significant difference in baseline DBP between patients which might lead to difficulty to appreciate the effect of DBP on postoperative orthostasis. From a physiologic point of view, reduced arterial pressure will lead to reduced cardiac output and associated with reduced cerebral blood flow and oxygenation and finally lead to symptoms of OI during upright position [5].

In our study high amount of intraoperative blood loss is associated with postoperative OI. This finding is in contrary with previous studies in which intraoperative blood loss is not associated OI [5, 8, 28]. The reason for discrepancy might be due to the inclusion of large sample size and diverse group of patients and surgical procedures including emergency and major surgeries which leads to significant and variable intraoperative blood loss. This finding showed that the etiology of post-operative OI is multifactorial. High amount of intraoperative blood loss leads to reduction in cardiac output and central blood volume which leads to reduction in cerebral blood flow and oxygenation and causes orthostasis during postoperative mobilization and upright position.

In our study types of anesthesia have been found significantly associated with the prevalence of postoperative OI. Patients who received general anesthesia have more likelihood of postoperative OI than those who take regional anesthesia. It was supposed that the association might be due to the effect of the various anesthetic drugs, and other perioperatively used drugs that lead to baroreflex dysfunction and impaired cardiac and reflex vasoconstriction control mechanism [11, 31].

In our study, pain scores during rest and during the time of first ambulation were considerably higher with median (IQR) 4 (2–6) and 7 (6 to 8) respectively and associated with postoperative OI. Similar to our study, higher pain score during ambulation is significantly associated with postoperative OI in study conducted in New Zealand [27]. In contrast to our study pain score during rest is not associated with postoperative OI in previous studies conducted to assess effect of pain in relation to postoperative OI [5, 22]. The difference for this finding may be due to the use of standardized pain treatment protocol in previous studies which may lead to difficulty to suggest pain score difference between orthostatic tolerant and intolerant individuals. However, from a physiological viewpoint, strong nociceptive input from painful stimuli can directly activate the medullary cardiovascular centers in the hypothalamus and lead to activation of vasovagal response. In addition to this, high pain may also be associated with fear and anxiety which further triggers the vasovagal response and may in turn contributes to postoperative OI [11, 27].

The result of the present study also showed that high amount of postoperative fluid intake is significantly associated with postoperative OI. Similar to our study, the study conducted in hip and knee arthroplasty patients showed that postoperative fluid intake is significantly higher in patients with OI than non-OI patients [25]. In contrary to our study, postoperative fluid intake is not associated with OI in studies conducted on patients with laparoscopic colorectal resection in Denmark and hip arthroplasty patients in New Zealand [27, 28]. This finding of our study is in contrary the idea which states that postoperative OI is caused by perioperative fluid loss and volume depletion. However, as different studies stated, administration of large volumes of fluid can lead to reduced baroreceptor sensitivity and can cause reflex hypotension [32–34]. The inhibition of baroreflex center due to volume overload might increase the risk of OI. Taking all the arguments in the current and the previous studies into consideration, further investigations that involve high-level studies are recommended.

Finally this study is not free of limitations. First, this study represents data from one institution and generalizability may be difficult. Secondly, during data collection in some of the participants the symptoms of orthostatic intolerance were asked retrospectively which may cause recall bias.

## Conclusion

The finding of our study revealed that postoperative OI was experienced in nearly two-thirds of study participants in University of Gondar Comprehensive Specialized Hospital. Female sex, low BMI, low preoperative DBP, ASA physical status II and above, general anesthesia, high intraoperative blood loss, high amount of postoperative fluid, pain during at rest and during ambulation were factors significantly associated with postoperative OI. So, maintaining preoperative normal blood pressure, minimizing intraoperative blood loss through different blood conservation strategies and managing postoperative pain adequately may help to reduce the risk of postoperative OI. During the time of ambulation, assessing patients for the presence of OI is necessary to reduce the risk of fall down injury and severe trauma. Additionally, more emphasis should be given to risky surgical patients and for these groups of patients we suggest specific postoperative mobilization protocols guided by clinicians and caregivers to be followed to reduce the adverse effects of postoperative OI.

Although the current study has come up with evidence revealing that high amount of postoperative fluid intake increases the risk of postoperative OI, it contradicts the existing literature and it is quite impossible to draw a conclusion based on this study. Therefore, future scholars are recommended to conduct a study that helps solve the observed contradiction.

## Data Availability

The data sets used and analyzed during the study are available from the corresponding author on reasonable request.

## Abbreviations

ASA: American Society of Anesthesiologist
AOR: Adjusted Odds Ratio
CI: Confidence Interval
COR: Crude Odds Ratio
DBP: Diastolic Blood Pressure
DM: Diabetes Mellitus
EBL: Estimated Blood Loss
ERAS: Enhanced Recovery After Surgery
NRS: Numeric Rating Scale
OI: Orthostatic Intolerance
SBP: Systolic Blood Pressure
UOGCSH: University of Gondar Comprehensive Specialized Hospital
